# Evolutionary game study on the governance and development of online car-hailing based on blockchain technology

**DOI:** 10.1038/s41598-022-11741-4

**Published:** 2022-06-07

**Authors:** Xiaoyu Wan, Jia Liu, Siqi Zhao

**Affiliations:** School of Economics and Management, Chongqing University of Posts and Telecommunications, Chongqing, 400065 China

**Keywords:** Applied mathematics, Software, Socioeconomic scenarios, Sustainability

## Abstract

Changes in the online car-hailing industry have brought new challenges to government governance. Effectively enhancing governance efficiency has become the focus of academic research. Based on the technical governance perspective, this paper introduces the consortium blockchain to construct an evolutionary game model between the online car-hailing platform and the government under blockchain technology. By solving the replicated dynamic equations and the Jacobian matrix, the influences of the change in initial conditions and decision parameters on the evolutionary stability results are revealed, and numerical experiments are carried out by using the Python programming language. This paper claims that the system presents three evolutionary stable results and a periodic stochastic state when the key parameters are located in different thresholds. The additional cost of the platform’s negative regulation and the government’s punishment intensity have a positive effect on the evolution of the system to the ideal state (active regulation, active governance). Platform technology R&D cost and government innovation input have a negative effect on the evolution of the system to the ideal state. Therefore, using blockchain to increase the additional cost of the platform’s negative regulation, appropriately increasing the government’s punishment intensity, reasonably controlling the government’s innovation input to the platform, and reducing the technology R&D cost of the platform will help the system evolve into an ideal state. This paper provides useful references to implement effective governance and the innovative and healthy development of the online car-hailing industry.

## Introduction

As a product of the integration of traditional travel and next-generation information technology, online car-hailing has been rapidly developed by satisfying customers’ personalised travel needs at a lower cost^1^. It has become one of the best choices for public travel. In November 2021, a total of 255 online car-hailing platforms in China were licenced for operation^2^. In December 2021, the number of online car-hailing customers in China reached 453 million^3^. The online car-hailing industry has shown explosive growth, with great development prospects and market value. However, the potential safety hazards of the online car-hailing industry also frequently occur^4–7^. From the customer perspective, drivers and passengers may face moral hazard, opportunism and other issues due to asymmetric information and incomplete contracts^8,9^. From a platform perspective, a centralised online car-hailing platform may have potential unreliable issues, such as illegally tampering with data and illegally collecting users’ privacy^10^. Therefore, the regulation and governance of the online car-hailing industry has become the focus of academic research.

Many scholars have carried out research on the regulation and governance of online car-hailing and have presented multiple paths of online car-hailing governance from the perspectives of legal norms, public management, and governance model design. Specific governance modes include policy and regulation formulation^11,12^, experimental regulation^13^, third-party regulation^14^, government-led regulation^6^, platform self-regulation^15,16^, and multi-subject collaborative governance^8,9,15^. These governance modes have effectively regulated the online car-hailing industry to a certain extent. However, the existing research is more focused on suggestions from the perspective of governance by participants and few scholars propose solutions from the perspective of technical governance. The online car-hailing industry was created on the internet, and its operation and development are based on internet technology. Therefore, it is necessary to explore how to improve the governance efficiency of the online car-hailing industry from a technical perspective. At the same time, the centralisation problem of online car-hailing is serious, resulting in information asymmetry between the platform, the government and the customers. The complaint channel of customers is not smooth, and the feedback is not timely. All of these factors will affect the governance effect of the platform^4,6,17^.

Facing the regulatory dilemma of online car-hailing, the development of blockchain technology injects new vitality into its governance work^18,19^. With the advantages of decentralisation, openness, transparency, cryptographic property, tamper-proofing and immutability, blockchain technology can realise functions, such as depository, credit enhancement and authentication, providing security for the collaboration of transaction parties^20,21^. Building a consortium blockchain composed of an online car-hailing platform, drivers, customers and government regulators enables safe and credible data sharing among participants, thereby reducing information asymmetry among all parties. Ultimately, by providing technical credit enhancement support for the online car-hailing industry, blockchain resolves the trust issues of multiple stakeholders^22–24^.

Based on the technical governance perspective, blockchain technology is introduced as the governing measure of online car-hailing. This paper constructs an evolutionary game model between the online car-hailing platform and the government through blockchain by using evolutionary game theory and correlated knowledge and discusses the factors that influence the strategic choices of online car-hailing platform regulation and government governance within the blockchain technology environment. On the basis of improving the level of government governance, the innovative and healthy development of the online car-hailing industry can be realised. This paper makes the following contributions. (1) From a technical governance perspective, this paper reveals the internal mechanism of behaviour evolution between the platform and the government to a certain extent and verifies the rationality of the results through numerical experiments. (2) This paper shows that the introduction of blockchain technology can effectively improve the governance efficiency of the online car-hailing industry, which provides new ideas for the governance and the development of this industry.

The remainder of this paper is structured as follows. “Literature reviews” reviews the related literature. “Model construction” constructs an evolutionary game model between the online car-hailing platform and the government through no-blockchain technology. “Blockchain-based model construction” constructs an evolutionary game model between the online car-hailing platform and the government through blockchain technology and conducts numerical simulation based on the model analysis. “Results and discussion” summarises the main findings and makes corresponding recommendations. “Conclusions” presents the conclusion.

## Literature reviews

### Online car-hailing governance

Online car-hailing is an emerging form of the sharing economy that uses idle vehicles to accept orders through the internet to provide travel services. To promote the healthy and sustainable development of the online car-hailing industry, scholars have discussed the regulation and governance of online car-hailing. Some scholars have suggested that though affected by the traditional taxi industry regulatory model, the governance measures of the Chinese government are relatively simple, and the governance framework is not suitable for the emerging online car-hailing industry^15,16,25^. This is mainly reflected in the deviations in the formulation and implementation of regulatory policies^26^. Therefore, government regulators need to explore more flexible and diverse regulation measures and improve governance resources, channels and links to achieve a good connection with the regulation of online car-hailing platforms^13,17,18^.

With the theme of improving the regulatory governance system, scholars have carried out analyses based on different research perspectives. With the goal of minimising welfare loss, Zhen constructed a control model for the quantity and price of online car-hailing based on two-sided market theory and concluded that the quantity control policy and the price control policy can effectively reduce the negative externalities of the online car-hailing industry^11^. In this regard, Wang asserted through theoretical analysis that quantity control is not in line with the purpose of saving social resources under the sharing economy model, and it is not appropriate to limit price control when the car level is high and the customer’s willingness to pay is high. The cooperative regulation mode of “the government governs the platform, and the platform regulates drivers and vehicles” can achieve more effective governance^27^. Li et al. verified the feasibility of this cooperative regulation model by constructing a tripartite game between the government, the online car-hailing platform and the taxi industry^28^. To better realise collaborative regulation, some scholars have claimed that government regulators should take the opportunity of the three-stream coupling of politics, policy and problem, comprehensively consider factors, such as the government's multiple motivations, customer service demand and the interest of the taxi industry, and finally carry out policy innovations according to local and time conditions^12,29,30^. After that, some scholars supplemented the regulation system. Wang et al. used process-tracking methods to sort out and summarise the development process of China’s online car-hailing industry from 2014 to 2018 and proposed an agile regulatory model characterised by a “challenge-response”^6^. Fu and Shi constructed an evolutionary game model between government regulators and the online car-hailing platform and verified the effectiveness of third-party regulation by introducing media coverage^14,31^. By sorting out the representative studies on online car-hailing governance (see Table 1), it was found that the existing research mainly focuses on theoretical analysis, and there are relatively few studies on quantitative analysis of online car-hailing governance based on mathematical models. In addition, previous studies have proposed solutions from the perspective of governance by participants, and few scholars have carried out research on online car-hailing governance from the perspective of technical governance.

**Table 1:** The representative studies on online car-hailing governance.

Authors	Research method	Research perspective	Core idea/governance model
Wang et al.^6^	Case study	Governance model design	A government-led “challenge-response” agile regulatory model
Zhuo and Wang^8^	Qualitative analysis and quantitative analysis	Normative mechanism design	Design information mechanisms and rules on the basis of government-platform collaborative regulation model
Lei et al.^9^	Mathematical modelling and evolutionary game theory	Governance model design	A multi-subject collaborative co-governance model including multinational transportation network company, driver and passenger
Zhen^11^	Mathematical modelling	Legal norms	The quantity and price control policies of online car-hailing
Huang and Li^12^	Policy text analysis and case study	Legal norms	This paper provides a useful reference for the formulation of online car-hailing regulation policies
Zhang^13^	Theoretical analysis	Public management and legal norms	An experimental regulatory model
Fu and Shi^14^	Mathematical modelling and evolutionary game theory	Governance model design	Third-party regulation model
Ren and Shen^15^	Theoretical analysis	Governance model design	A dynamic cooperative regulation system combining government-platform cooperative regulation and platform self-regulation
Guo^16^	Theoretical analysis	Governance model design and legal norms	This paper proposes the platform self-discipline regulation model and the multi-subject collaborative co-governance model, and emphasises the need for the government to reform inappropriate laws and regulations
Wang^27^	Theoretical analysis	Governance model design	A cooperative regulation model of “the government governs the platform, and the platform regulates drivers and vehicles”
Cao^29^	Theoretical analysis	Public management	This paper emphasises the importance of keeping pace with the times and adapting to local conditions
This paper	Mathematical modelling and evolutionary game theory	Technology governance and governance model design	The blockchain technology governance model and government-platform collaborative co-governance model

### Blockchain technology

To solve the trust issues in network transactions, Satoshi Nakamoto developed and proposed blockchain technology. It is a digitalised ledger that integrates technologies such as consensus algorithms, encryption algorithms and smart contracts, which record transaction data in block form in chronological order and store them in a distributed manner in the network^32^. As a distributed encrypted database with a decentralised consensus mechanism, blockchain combines the technical characteristics of openness, transparency, security and reliability, traceability, decentralisation, tamper-proofing and immutability^23,33^. It guarantees secure transactions between untrusted nodes or parties by providing a complete security solution^20^. According to the differences in data management and read permissions of transaction subjects, blockchain can be divided into public blockchain, private blockchain and consortium blockchain^34^. Among them, the public blockchain has no central authority, and anyone can participate in the process of obtaining consensus^32^. The private blockchain is created by a single organisation or individual, the management permission is strictly controlled by the creator, and the read permission is finitely open to the public^34^. The consortium blockchain is an amalgamation of public and private blockchains. It is composed of multiple organisations with cooperative relationships. The permissions of members are formulated according to the rules of the consortium^32,34^. This paper is dedicated to introducing a consortium blockchain that is jointly maintained by the online car-hailing platform, drivers, customers and government regulators, which can not only meet the needs of different participants but also meet the regulatory requirements of all parties. Due to its unique technological advantages, blockchain has been widely used by scholars to conduct in-depth research in many fields, such as smart cities^20^, sharing economy^23^, public administration^35^, health care^36^ and energy trading^37^. Almost all industries need an honest, reliable and trustworthy environment as a prerequisite for healthy and sustainable development. Blockchain creates trust for transaction subjects through mathematical principles rather than using third-party regulation. By maintaining the security, transparency and consistency of the ledgers among the subjects, the information asymmetry of the participants can be effectively reduced^16,17,37^.

### Evolutionary game theory

Evolutionary game theory originated from Darwin's theory of evolution, which emphasises the bounded rationality of game players and the dynamic changes of the game process. Specifically, it means that players with bounded rationality cannot reach a stable equilibrium state at one time. They need to learn by replicated dynamic mechanisms and undergo long-term dynamic adjustments, eventually reaching an equilibrium state during evolution^38,39^. Evolutionary game theory can truly reflect the diversity and complexity of the behaviour of economic agents^38^, so it is widely used in the field of multi-participant collaborative governance. Zhou et al. constructed and discussed a tripartite evolutionary game model of sewage enterprises, governments and the public, and made suggestions for environmental pollution control^40^. Yang et al. constructed an evolutionary game model composed of local government, food enterprises and the public for analysis, aiming to reduce the hidden safety issues in the food market^41^. Qi et al. constructed an evolutionary game model between the “Internet Big V” and the official media and then introduced a punishment mechanism and coordination mechanism for government departments, aiming to realise the proper handling of emergencies and the effective governance of network public opinion^42^. Evolutionary game theory has been a part of many governance studies in various fields, but there are few studies on the governance and development of online car-hailing based on evolutionary game theory. In essence, the governance of online car-hailing is a dynamic game problem that involves multiple parties and evolves over time. Participants constantly adjust their strategies through confrontation, dependence and constraints before finally reaching a state of equilibrium^43^. Therefore, evolutionary game theory provides a scientific analysis paradigm for this paper. In this paper, it is necessary to analyse the interaction mechanism of government governance and platform regulation from the perspective of dynamic evolution and to reveal the universal laws of the governance and the development of the online car-hailing industry.

## Model construction

### Model assumptions

In this paper, we consider that platforms and governments are bounded rational, and they usually make incomplete rational strategic choices through incomplete information^44^. In the context of incomplete information, the platform can choose active regulation, meaning that the platform consciously abides by industry regulations, strictly scrutinises illegal drivers, and makes full use of the blockchain to build a trust system. The platform can also choose negative regulation, meaning that the platform is not strict in the scrutinisation of driver qualifications and vehicle standards. The platform negatively responds to the regulatory responsibilities and only addresses problems superficially. The government can choose active governance, meaning that the government fines the platform for negative regulation. The government provides technical subsidies and formulates preferential policies to encourage the healthy development of the online car-hailing industry. The government can also choose negative governance, meaning that the government regulator has neglected its duty and has not strictly dealt with the illegal behaviour of the platform. The government does not provide financial subsidies for innovative behaviours in the online car-hailing industry. On this basis, this paper employs an evolutionary game to analyse the payoff matrix for both players, where the strategic space for platform is (active regulation $x$, negative regulation $1-x$) and for government is (active governance $y$, negative governance $1-y$).

Hypothesis 1. The basic revenue of the platform is $R$. When the platform chooses active regulation, it pays operating cost ${C}_{2}$ (the cost of scrutinising driver qualifications and vehicle standards, etc.), and generates social welfare $S$ for the public to create a safe and civilised travel environment. When the platform chooses negative regulation, it causes social welfare losses $L$ (the loss of customer benefits and governance benefits and impacts other industries, etc.). For this, the government that chooses active governance imposes fine $f$ on the platform, and $f> {C}_{2}$; otherwise, the platform has no incentive to choose active regulation.

Hypothesis 2. When the government chooses active governance, it invests a great deal of governance cost ${C}_{1}$ (manpower, time and material costs, etc.) to strictly control and rectify the online car-hailing industry, and ${C}_{1}>f$. The governance cost is higher than the fine imposed on the platform that chooses negative regulation.

On this basis, we derive the payoff matrix of the platform and the government, as presented in Table 2.

**Table 2:** The payoff matrix of the platform and the government.

Online car-hailing platform	Government	
	Active governance ($y$)	Negative governance ($1-y$)
Active regulation ($x$)	$R-{C}_{2}$, $S-{C}_{1}$	$R-{C}_{2}$, $S$
Negative regulation ($1-x$)	$R-f$, $f-{C}_{1}-L$	$R$, $-L$

### Evolutionary stability analysis

According to Table 2, when the platform chooses active regulation, the expected revenue ${E}_{x1}$ is as follows:

1 $${E}_{x1}=y\left(R-{C}_{2}\right)+\left(1-y\right)\left(R-{C}_{2}\right)=R-{C}_{2}$$

When the platform chooses active regulation, the expected revenue ${E}_{x2}$ is as follows:

2 $${E}_{x2}=y\left(R-f\right)+\left(1-y\right)R=R-yf$$

The average expected revenue $\overline{{E }_{x}}$ of the platform is as follows:

3 $$\overline{{E }_{x}}=x{E}_{x1}+\left(1-x\right){E}_{x2}=R-x{C}_{2}-yf+xyf$$

Similarly, the average expected revenue $\overline{{E }_{y}}$ of the government is as follows:

4 $$\overline{{E }_{y}}=y{E}_{y1}+\left(1-y\right){E}_{y2}=yf-y{C}_{1}-xyf+xS+xL-L$$

According to Malthusian dynamic equation theory, we obtain the replicated dynamic equations of the platform and the government, and they are calculated as follows:

5 $$\left\{\begin{array}{c}F\left(x\right)=\frac{{d}_{x}}{{d}_{t}}=x\left({E}_{x1}-\overline{{E }_{x}}\right)=x\left(1-x\right)\left(yf-{C}_{2}\right)\\ F\left(y\right)=\frac{{d}_{y}}{{d}_{t}}=y\left({E}_{y1}-\overline{{E }_{y}}\right)=y\left(1-y\right)\left(f-xf-{C}_{1}\right)\end{array}\right.$$

We calculate the replicated dynamic equations of the platform and the government when $F\left(x\right)=0$ and $F\left(y\right)=0$. Then, the five local equilibrium points of the platform and the government strategic choices can be calculated as ${N}_{1}\left(0 , 0\right)$, ${N}_{2}\left(0 , 1\right)$, ${N}_{3}\left(1 , 0\right)$, ${N}_{4}\left(1 , 1\right)$, and ${N}_{5}\left({x}^{*} , {y}^{*}\right)$, where ${x}^{*}=\frac{f-{C}_{1}}{f}$ and ${y}^{*}=\frac{{C}_{2}}{f}$.

The local equilibrium point calculated by the replicated dynamic equation is not necessarily the evolutionarily stable result of the game system. Therefore, according to the Jacobian matrix solution method in Friedman^43^, we calculate the Jacobian matrix of the game system as follows:

6 $$J=\left[\begin{array}{cc}\frac{\partial F(x)}{\partial x}& \frac{\partial F(x)}{\partial y}\\ \frac{\partial F(y)}{\partial x}& \frac{\partial F(y)}{\partial y}\end{array}\right]=\left[\begin{array}{cc}\left(1-2x\right)\left(yf-{C}_{2}\right)& x(1-x)f\\ -y(1-y)f& (1-2y)(f-xf-{C}_{1})\end{array}\right]$$

When the Jacobian matrix meets $detJ>0$ and $trJ<0$, the local equilibrium point of the replicated dynamic equation is the ESS point. At the same time, since the solution of the evolutionary game is a strict Nash equilibrium in this paper, we only discuss the first four points. The results of evolutionary stability analysis of the platform and the government are shown in Table 3.

**Table 3:** Stability analysis of local equilibrium points.

Local equilibrium point	$detJ$	$trJ$	Stability
${N}_{1}\left(0 , 0\right)$	+	−	ESS
${N}_{2}\left(0 , 1\right)$	+	+	Unstable
${N}_{3}\left(1 , 0\right)$	−	−	Unstable
${N}_{4}\left(1 , 1\right)$	−	+	Unstable

According to Table 3, without the introduction of blockchain, only one local equilibrium point in the game system between the platform and the government is stable. That is, N_1_(0,0) is the only ESS point. The implication of this ESS is that the online car-hailing platform chooses negative regulation and the government chooses negative governance. This strategy is not conducive to the healthy development of the online car-hailing industry and is a negative state of mutual inaction between the platform and the government.

## Blockchain-based model construction

### Model assumptions

**Hypothesis 1.** Blockchain technology can improve the safety, synergy and efficiency of online car-hailing, and further enhance the efficiency of urban transportation systems. According to the innovation maturity theory proposed by Jay Paap and Ralph Katz^45^, we set technical efficiency as $P=T{C}_{3}$, where $T$ is the blockchain technology maturity, and ${C}_{3}$ is the R&D investment for the platform to introduce blockchain technology.

**Hypothesis 2.** The basic revenue of the platform is *R*. When the platform chooses active regulation, it pays operating cost ${C}_{2}$ and technology R&D cost ${C}_{3}$ and generates social welfare $S$; when the platform chooses negative regulation, it causes social welfare losses $L$. In the case of the introduction of blockchain, the platform's negative regulation pays high additional cost $d$ (the cost of data tampering and information fabrication, loss of reputation, etc.).

**Hypothesis 3.** When the government chooses active governance, it pays total governance cost $C$ consisting of fixed governance cost and marginal governance cost. Blockchain technology reduces the risk of information asymmetry by reducing the probability of information distortion, thereby effectively reducing the cost of governance. Therefore, blockchain technology has a certain degree of substitution with governance. The higher blockchain technology maturity is, the lower the marginal governance cost is. We set the total governance cost as $C={C}_{1}+(1-T)/k$, where ${C}_{1}$ is the fixed governance cost and $k$ is a constant representing the governance capacity.

**Hypothesis 4.** When the government chooses active governance, it encourages the online car-hailing industry to introduce blockchain for innovative development by increasing investment, providing financial subsidies, and introducing relevant policies. These inputs are set as government innovation input $F$. In addition, the government imposes fine $f$ on the platform that chooses negative regulation.

On this basis, we derive the payoff matrix of the platform and the government under blockchain technology, presented in Table 4.

**Table 4:** The payoff matrix of the platform and the government under blockchain technology.

Online car-hailing platform	Government	
	Active governance ($y$)	Negative governance ($1-y$)
Active regulation ($x$)	$R+P+F-{C}_{2}-{C}_{3}$, $S-F-C$	$R+P-{C}_{2}-{C}_{3}$*,* $S$
Negative regulation ($1-x$)	$R+F-f-d$, $f-F-L-C$	$R-d$*,* $-L$

### Evolutionary stability analysis

According to Table 4, when the platform chooses active regulation, the expected revenue ${E}_{x1}$ is as follows:

7 $${E}_{x1}=y\left(R+P+F-{C}_{2}-{C}_{3}\right)+\left(1-y\right)\left(R+P-{C}_{2}-{C}_{3}\right)=yF+R+P-{C}_{2}-{C}_{3}$$

When the platform chooses active regulation, the expected revenue ${E}_{x2}$ is as follows:

8 $${E}_{x2}=y\left(R+F-f-d\right)+\left(1-y\right)\left(R-d\right)=yF-yf+R-d$$

The average expected revenue $\overline{{E }_{x}}$ of the platform is as follows:

9 $$\overline{{E }_{x}}=x{E}_{x1}+\left(1-x\right){E}_{x2}=xP-x{C}_{2}-x{C}_{3}+y\alpha F-yf+R-d+xyf+xd$$

Similarly, the average expected revenue $\overline{{E }_{y}}$ of the government is as follows:

10 $${E}_{y1}=x\left(S-F-C\right)+\left(1-x\right)\left(f-F-L-C\right)=xS+f-F-L-C-xf+xL$$

The replicated dynamic equations of the platform and the government obtained by calculation are as follows:

11 $$\left\{\begin{array}{c}F\left(x\right)=\frac{{d}_{x}}{{d}_{t}}=x\left({E}_{x1}-\overline{{E }_{x}}\right)=x\left(1-x\right)\left(P+yf+d-{C}_{2}-{C}_{3}\right)\\ F\left(y\right)=\frac{{d}_{y}}{{d}_{t}}=y\left({E}_{y1}-\overline{{E }_{y}}\right)=y\left(1-y\right)\left(f-xf-C-F\right)\end{array}\right.$$

We calculate the replicated dynamic equations of the platform and the government when $F\left(x\right)=0$ and $F\left(y\right)=0$. Then, the five local equilibrium points of the platform and the government strategic choices can be calculated as ${M}_{1}\left(0 , 0\right)$, ${M}_{2}\left(0 , 1\right)$, ${M}_{3}\left(1 , 0\right)$, ${M}_{4}\left(1 , 1\right)$, and ${M}_{5}\left({x}_{0} , {y}_{0}\right)$, where ${x}_{0}=\frac{f-C-F}{f}$ and ${y}_{0}=\frac{{C}_{2}+{C}_{3}-d-T{C}_{3}}{f}$.

#### Platform evolutionary stability analysis

**Figure 1:**
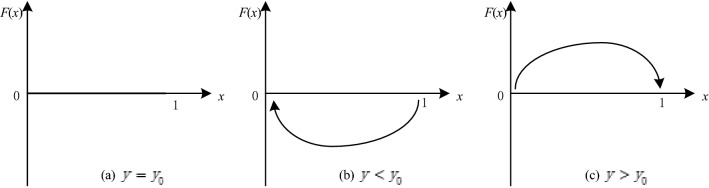
The dynamic trend of the platform's strategy.

The choice of evolutionary stability strategy usually depends on the initial state of both players. When $y={y}_{0}$, $x$ is stable at any level of $0\le x\le 1$, as shown in Fig. 1a. When $y\ne {y}_{0}$, ${x}^{*}=0$ and ${x}^{*}=1$ are stable. When ${x}^{*}$ satisfies ${F}^{^{\prime}}\left(x\right)<0$, ${x}^{*}$ is an evolutionarily stable strategy. Therefore, when $y<{y}_{0}$, $x=0$ is stable. When the governance is too low, the platform chooses negative regulation, as shown in Fig. 1b. When $y>{y}_{0}$, $x=1$ is stable, meaning that when governance reaches a certain level, active regulation is the optimal strategy for the platform in the game system, as shown in Fig. 1c.

#### Government’s evolutionary stability analysis

**Figure 2:**
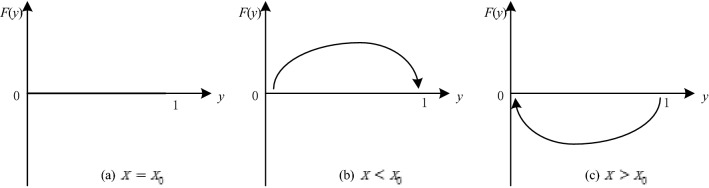
The dynamic trend of the government's strategy.

When $x={x}_{0}$, $y$ is stable at any level of $0\le y\le 1$, as shown in Fig. 2a. When $x\ne {x}_{0}$, ${y}^{*}=0$ and ${y}^{*}=1$ are stable. When ${y}^{*}$ satisfies ${F}^{^{\prime}}\left(y\right)<0$, ${y}^{*}$ is an evolutionarily stable strategy. Therefore, when $x<{x}_{0}$, $y=1$ is stable. Additionally, when the probability that the platform chooses active regulation is too low, active governance is the optimal strategy of the government in the game system, as shown in Fig. 2b. When $x>{x}_{0}$ and $y=0$ is stable. When the probability that the platform chooses active regulation reaches a certain level, the government eventually chooses negative governance, as shown in Fig. 2c.

#### Evolutionary path analysis

According to the Jacobian matrix solution method, we calculate the Jacobian matrix of the game system as follows:

18 $$J=\left[\begin{array}{cc}(1-2x)(T{C}_{3}+yf+d-{C}_{2}-{C}_{3})& x(1-x)f\\ -y(1-y)f& (1-2y)(f-xf-C-F)\end{array}\right]$$

Then, we calculate the determinant $(detJ)$ and trace $(trJ)$ of the Jacobian matrix, and the calculation results are shown in Table 5.

**Table 5:** The values of $detJ$ and $trJ$ corresponding to each equilibrium point.

Equilibrium point	$detJ$	$trJ$
${M}_{1}\left(0 , 0\right)$	$(T{C}_{3}+d-{C}_{2}-{C}_{3})(f-C-F)$	$\left(T{C}_{3}+d-{C}_{2}-{C}_{3}\right)+(f-C-F)$
${M}_{2}\left(0 , 1\right)$	$-(T{C}_{3}+f+d-{C}_{2}-{C}_{3})(f-C-F)$	$\left(T{C}_{3}+f+d-{C}_{2}-{C}_{3}\right)-(f-C-F)$
${M}_{3}\left(1 , 0\right)$	$(T{C}_{3}+d-{C}_{2}-{C}_{3})(C+F)$	$-\left(T{C}_{3}+d-{C}_{2}-{C}_{3}\right)-(C+F)$
${M}_{4}\left(1 , 1\right)$	$-(T{C}_{3}+f+d-{C}_{2}-{C}_{3})(C+F)$	$-\left(T{C}_{3}+f+d-{C}_{2}-{C}_{3}\right)+(C+F)$
${M}_{5}\left({x}_{0} , {y}_{0}\right)$	${x}_{0}{y}_{0}{f}^{2}(1-{x}_{0})(1-{y}_{0})$	$0$

When the Jacobian matrix meets $detJ>0$ and $trJ<0$, the equilibrium point of the replicated dynamic equation is the ESS point. According to Table 5, the game system has a saddle point $\left({x}_{0},{y}_{0}\right)$. The evolutionary stability result of the system changes with the different value ranges of ${x}_{0}$ and ${y}_{0}$. Among them, it is obviously not true when ${x}_{0}>1$. Therefore, we discuss the values of ${x}_{0}$ and ${y}_{0}$ in different situations.

1. When ${x}_{0}<0$ and ${y}_{0}<0$, $f-C-F<0$ and ${C}_{2}+{C}_{3}-d-T{C}_{3}<0$ so that $\left(1 , 0\right)$ is the ESS point of the system.
2. When ${x}_{0}<0$ and $0<{y}_{0}<1$, $f-C-F<0$ and $0<{C}_{2}+{C}_{3}-d-T{C}_{3}<f$,so that $\left(0 , 0\right)$ is the ESS point of the system.
3. When ${x}_{0}<0$ and ${y}_{0}>1$, $f-C-F<0$ and ${C}_{2}+{C}_{3}-d-T{C}_{3}>f$ so that $\left(0 , 0\right)$ is the ESS point of the system.
4. When $0<{x}_{0}<1$ and ${y}_{0}<0$, $f-C-F>0$ and ${C}_{2}+{C}_{3}-d-T{C}_{3}<0$ so that $\left(1 , 0\right)$ is the ESS point of the system.
5. When $0<{x}_{0}<1$ and $0<{y}_{0}<1$, then $f-C-F>0$ and $0<{C}_{2}+{C}_{3}-d-T{C}_{3}<f$ and there is no stable point in the system.
6. When $0<{x}_{0}<1$ and ${y}_{0}>1$, $f-C-F>0$ and ${C}_{2}+{C}_{3}-d-T{C}_{3}>f$ so that $\left(0 , 1\right)$ is the ESS point of the system.

**Figure 3:**
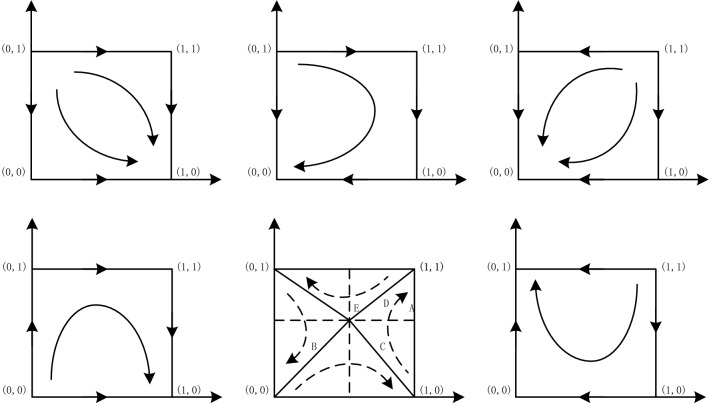
The evolutionary phase diagram of both players.

Based on the above discussion, the ESS points and the parameter conditions of the game system are shown in Table 6. The corresponding evolution phase diagrams for the six situations are shown in Fig. 3.

**Table 6:** ESS points and parameter conditions.

State	ESS point	The value of ${{\varvec{x}}}_{0}$ and ${{\varvec{y}}}_{0}$	Parameter condition
I	${M}_{1}\left(0 , 0\right)$	${x}_{0}<0$, ${y}_{0}>0$	$f-C-F<0$, ${C}_{2}+{C}_{3}-d-T{C}_{3}>0$
II	${M}_{2}\left(0 , 1\right)$	$0<{x}_{0}<1$, ${y}_{0}>1$	$f-C-F>0$, ${C}_{2}+{C}_{3}-d-T{C}_{3}>f$
III	Null	$0<{x}_{0}<1$, $0<{y}_{0}<1$	$f-C-F>0$, $0<{C}_{2}+{C}_{3}-d-T{C}_{3}<f$
IV	${M}_{3}\left(1 , 0\right)$	${x}_{0}<1$, ${y}_{0}<0$	${C}_{2}+{C}_{3}-d-T{C}_{3}<0$

#### Key parameter analysis

1. Key parameters related to the platform
  In states I and IV, the platform chooses negative regulation and active regulation, respectively. It is required to meet $\frac{\partial F(x)}{\partial x}<0$ so that the two states tend to be stable, and the conditions are $T{C}_{3}+d-{C}_{2}-{C}_{3}<0$ and $T{C}_{3}+d-{C}_{2}-{C}_{3}>0$, respectively. Among them, the platform technology R&D cost ${C}_{3}$ and the additional cost of passive regulation $d$ are important parameters that affect the platform. When the technology R&D cost is small and the additional cost is large, the expected revenue of the platform choosing active regulation is less than 0. For additional cost $d$, when the blockchain technology maturity is low, the platform chooses negative regulation to lose less, and the technical efficiency obtained by applying the blockchain is not enough to offset the governance cost of active regulation $({C}_{2}+{C}_{3})$. Thus, the probability of the platform choosing active regulation is low. For technical efficiency $P=T{C}_{3}$, since blockchain technology maturity $T$ is an uncontrollable parameter, to change the platform's strategy in the game system to an ideal state, the platform should not only make reasonable use of the government's innovation subsidies but also improve technology R&D efficiency, thereby reducing the technology R&D cost ${C}_{3}$.
2. Key parameters related to the government
  In states I and II, the government chooses negative governance and active governance, respectively. It is required to meet $\frac{\partial F(y)}{\partial y}<0$ so that the two states tend to be stable, and the conditions are $f-C-F<0$ and $f-C-F>0$, respectively. For total governance cost $C={C}_{1}+(1-T)/k$, since blockchain technology maturity $T$ is an uncontrollable parameter for the government, the key parameters for the establishment of $f-C-F>0$ are the government innovation input $F$ and punishment intensity $f$. When punishment intensity is small and innovation input is large, the expected revenue of the government choosing active governance is less than 0. When the platform chooses negative regulation, it brings high governance costs to the government, resulting in the net revenue of the government choosing that the active governance is less than 0, meaning that the punishment intensive is less than the sum of innovation input and governance cost. Driven by interests, the government tends to choose negative governance. At the same time, according to the conditions, government innovation input is not the larger the better. Therefore, to change the government's strategy in the game system to an ideal state, the government should not only increase the punishment intensity for the platform's negative regulatory behaviours, but also provide reasonable subsidies for the platform's application of blockchain to increase the willingness of the platform to apply blockchain technology without affecting the interests of the government, and finally achieve the optimal state of government governance.

### Numerical experiment

**Figure 4:**
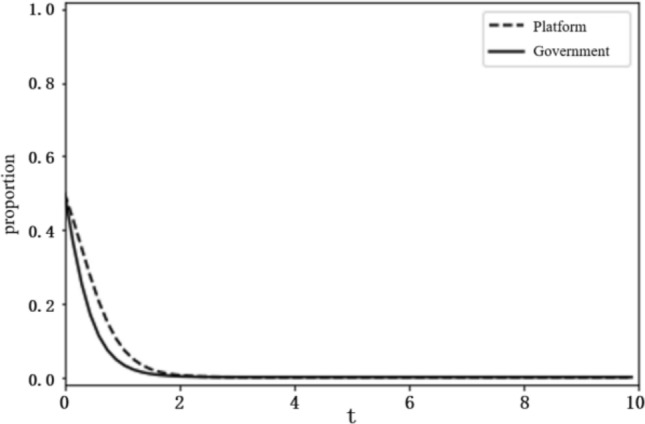
System evolution of ${C}_{3}=8$.

**Figure 5:**
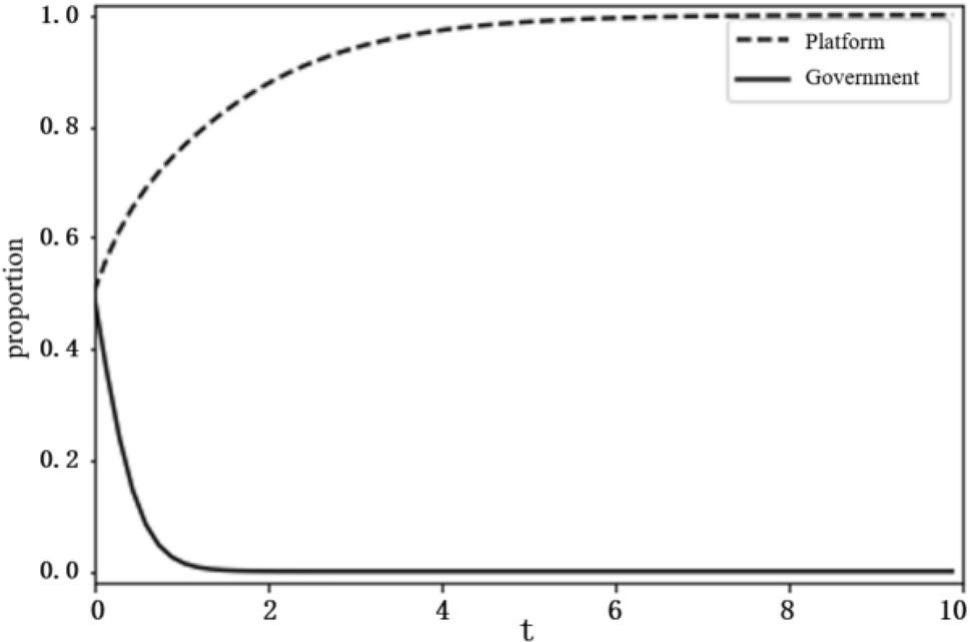
System evolution of ${C}_{3}=2$.

**Figure 6:**
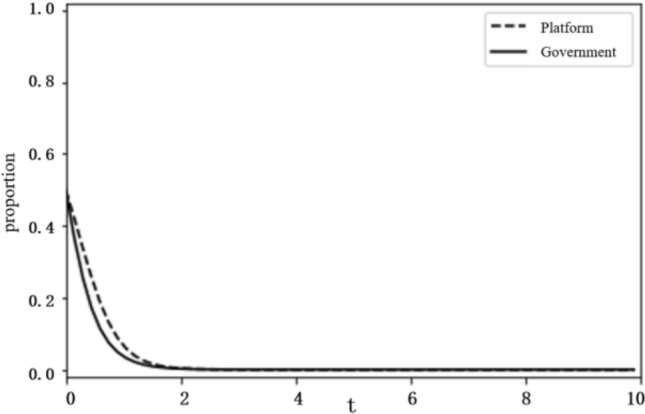
System evolution of $d=2$.

**Figure 7:**
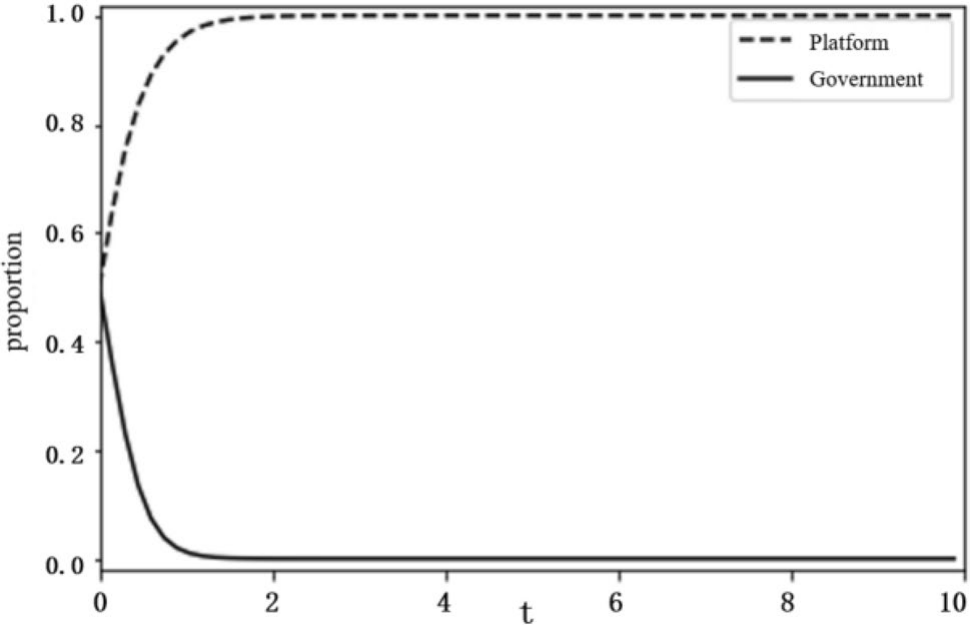
System evolution of $d=8$.

**Figure 8:**
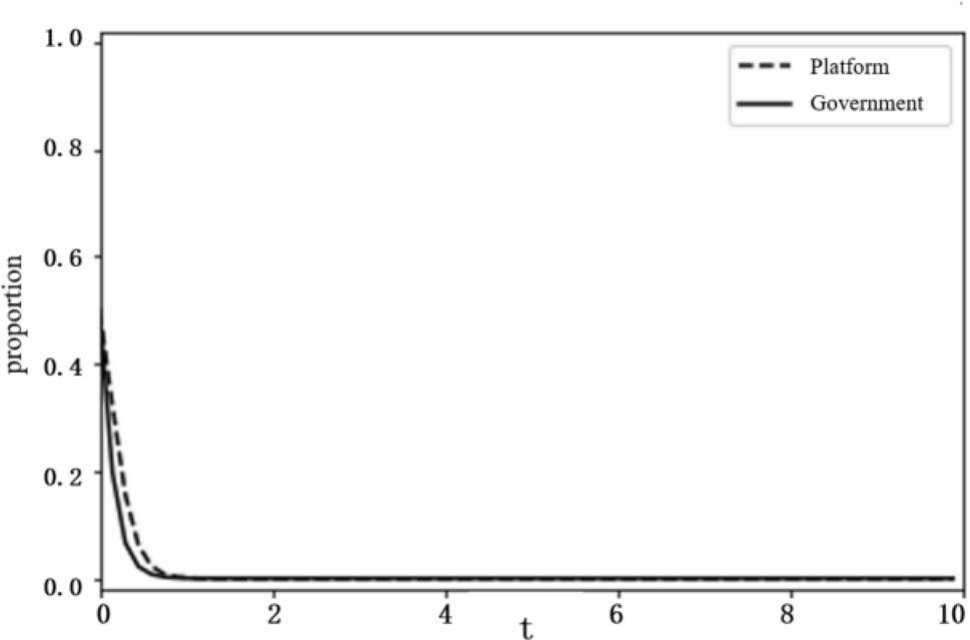
System evolution of $F=6$.

**Figure 9:**
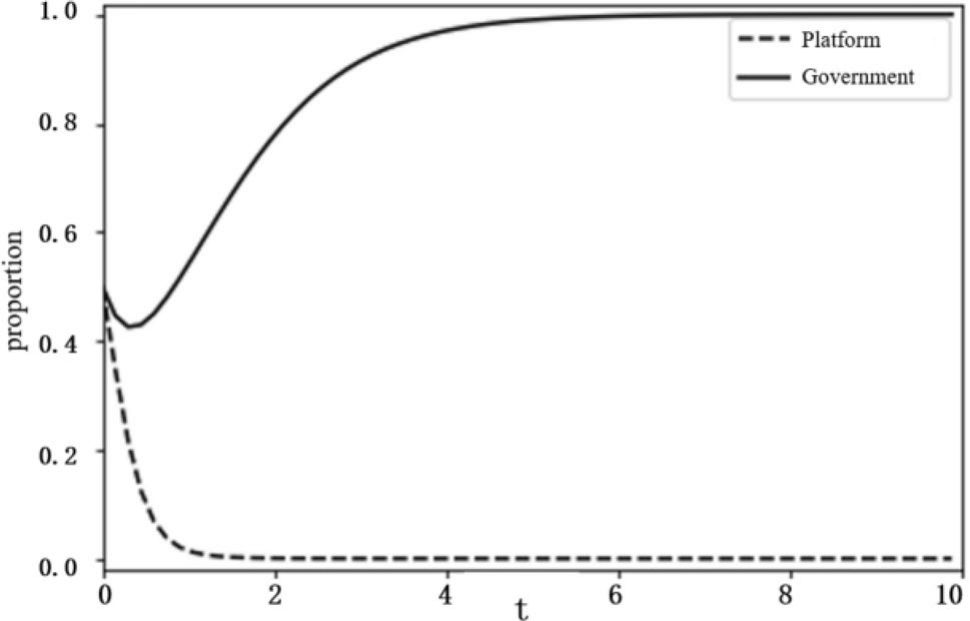
System evolution of $F=2$.

**Figure 10:**
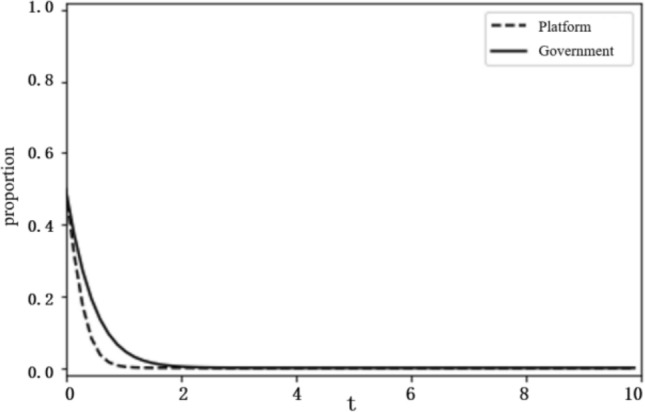
System evolution of $f=2$.

**Figure 11:**
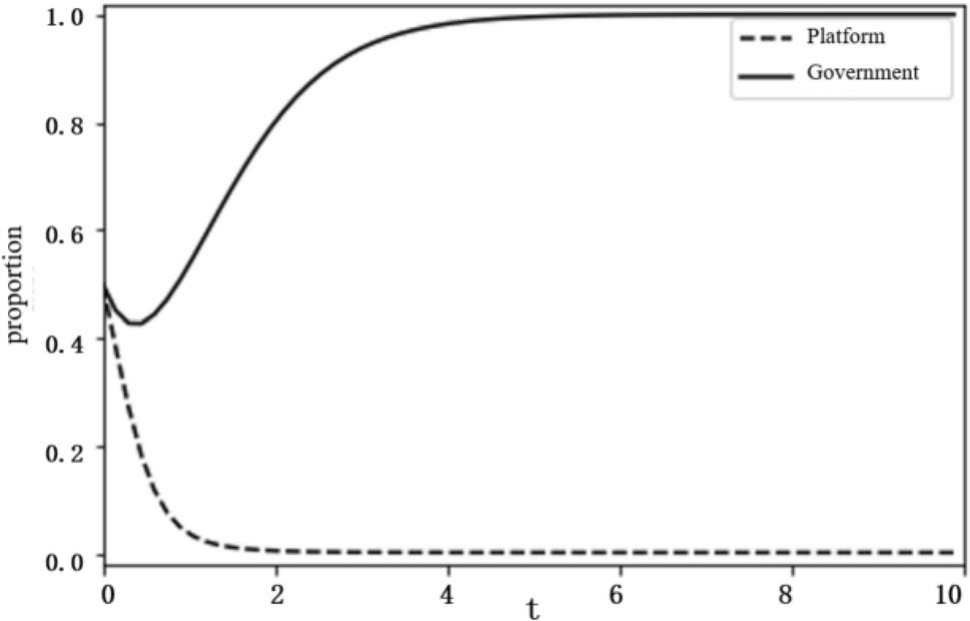
System evolution of $f=6$.

Based on the above analysis, the evolutionary stability results of the system depend on the initial conditions and changes in the relevant parameters. To more intuitively reflect on the evolutionary path of the game between the platform and the government and the influence of the parameter values on evolutionary stability results, this paper uses the Python programming language to carry out numerical experiments. The parameter initialisation values are shown in Table 7, and the system evolution diagrams are shown in Figs. 4, 5, 6, 7, 8, 9, 10 and 11. The horizontal axis in the figures represents the evolution time $t$ of the system, and the vertical axis represents the proportion $x(t)$ of the platform choosing active regulation and the proportion $y(t)$ of the government choosing active governance.

**Table 7:** Parameter initialisation values table.

**Parameter**	$f$	$d$	$T$	${C}_{1}$	${C}_{2}$	${C}_{3}$	$F$	$k$
Value	$6$	$1$	$0.4$	$2$	$4$	$5$	$2$	$0.8$

#### The impact of innovation input and punishment intensity

Technology R&D cost (${C}_{3}$). To make the system evolve to state IV, the condition ${C}_{2}+{C}_{3}-d-T{C}_{3}<0$ should be satisfied. We set the technology development cost ${C}_{3}$ as 2 and 8 and obtain the evolution curves of $x(t)$ and $y(t)$ following the change of ${C}_{3}$, as shown in Figs. 4 and 5. The figures show that when the technology R&D cost is relatively large, as ${C}_{3}=8$, the platform tends to choose negative regulation, and when the technology R&D cost is reduced to ${C}_{3}=2$, the platform turns to active regulation.

Additional cost ($d$). We set the additional cost $d$ as 2 and 8 and obtain the evolution curves of $x(t)$ and $y(t)$ following the change in d, as shown in Figs. 6 and 7. The figures show that when the additional cost is relatively small, as $d=2$, the platform tends to choose negative regulation, and when the additional cost increases to $d=8$, the platform turns to active regulation.

#### The impact of the technology R&D cost and the additional cost

Government innovation input ($F$). To make the system evolve to state $\mathrm{II}$, the conditions $f-C-F>0$, ${C}_{2}+{C}_{3}-d-T{C}_{3}>f$ should be satisfied. We set the government innovation input $F$ as 6 and 2, and obtain the evolution curves of $x(t)$ and $y(t)$ following the change of $F$, as shown in Figs. 8 and 9. The figures show that when innovation input is relatively large, as $F=6$, the government tends to choose negative governance, and when innovation input is reduced to $F=2$, the government turns to active governance.

Punishment intensity ($f$). We set the punishment intensity $f$ as 6 and 2 and obtain the evolution curves of $x(t)$ and $y(t)$ following the change of *f*, as shown in Figs. 10 and 11. The figures show that when punishment intensity is relatively small, as $f=2$, the government tends to choose negative governance, and when punishment intensity increases to $f=6$, the government turns to active governance.

## Results and discussion

To effectively improve the government governance efficiency and promote the innovative and healthy development of the online car-hailing industry, based on the perspective of technology governance, this paper introduces blockchain technology as the governance measure to construct an evolutionary game model between the online car-hailing platform and the government based on blockchain technology. This paper also analyses the evolutionary stability results and evolutionary paths of the system under different conditions. This paper draws the following results. (1) Government innovation input has a negative effect on the evolution of the system to an ideal state. Larger government subsidies for platform technology innovation are not better, and reasonable innovation input can prompt the government to choose active governance. (2) Government punishment intensity has a positive effect on the evolution of the system to an ideal state. Increasing the punishment intensity for negative regulatory behaviour of the platform can help motivate the government to choose active governance. (3) The technology R&D cost has a negative effect on the evolution of the system to an ideal state. Effectively reducing the platform's R&D cost for blockchain technology can prompt the platform to choose active regulation. (4) The additional cost has a positive effect on the evolution of the system to the ideal state. When the information fraud cost and reputation loss caused by the platform's negative regulation are high, it can help the platform choose active regulation.

According to the above results, to help the government better govern the online car-hailing platform and promote the innovation and development of the platform, this paper proposes the following countermeasures. From the perspective of government governance, the government should strengthen the risk assessment of innovation subsidies for the platform and provide appropriate subsidies to the platform that actively introduces blockchain technology for innovation and development. At the same time, the government should establish and perfect policies and regulations on the governance of the online car-hailing industry. On the basis of clarifying the platform's regulatory responsibilities, the government urges the platform to earnestly fulfil its regulatory responsibilities and imposes high fines on the platform that defrauds subsidies and negatively responds to the regulatory responsibilities. From the perspective of platform regulation, the platform should make reasonable use of government innovation subsidies, and actively improve the R&D efficiency of blockchain technology to effectively reduce the R&D cost of blockchain technology. At the same time, the platform should make full use of blockchain technology to create a trustworthy environment, which will send a safe and reliable signal to the public, thereby effectively enhancing the reputation of the platform and ultimately realising the innovative and healthy development of the online car-hailing industry.

## Conclusions

The governance and the development of the online car-hailing industry is an important measure to ensure a safe and civilised travel environment. With the technical characteristics of openness, transparency, security and reliability, traceability, decentralisation, tamper-proofing and immutability, blockchain technology provides technical credit enhancement support and secure transaction guarantees for the online car-hailing industry^14,17^. Therefore, based on evolutionary game theory, this paper introduces blockchain as a technical governance measure and constructs an evolutionary game model between the online car-hailing platform and the government based on blockchain technology. By establishing and solving the replicated dynamic equations of both players, the evolutionary equilibrium strategy of the participants and the stable state of the system are analysed. On this basis, focusing on the four key parameters of the government innovation input, punishment intensity, blockchain technology R&D cost and additional cost of the platform's negative regulation, we deeply analyse the impact of parameter changes on the strategic choices of both players, and finally use the Python programming language to verify the model’s results.

From the perspective of technology governance, this paper reveals the internal mechanism of the behaviour evolution between the online car-hailing platform and the government to a certain extent and verifies that the introduction of blockchain technology can effectively realise the regulation and governance of the online car-hailing industry. Finally, this paper provides a useful reference for the governance and the development of the online car-hailing industry from two levels of government governance and platform regulation. In addition, this paper has some limitations. First, due to the limited data collection, the numerical simulation lacks the support of real data. Future research can be combined with real cases in the online car-hailing industry to address the complex and the changeable market environment. Second, this paper only considers the two-dimensional game between the government and the platform. Future research can introduce more subjects, such as online car-hailing drivers and customers, and further explore how to achieve the governance and healthy development of the online car-hailing industry in the case of multiple participants.

## Data Availability

All data generated or analysed during this study are included in this published article.
